# Abscopal responses in patients with metastatic melanoma involving skin and subcutaneous tissues treated with intralesional IL2 plus BCG

**DOI:** 10.3389/fonc.2023.1160269

**Published:** 2023-04-27

**Authors:** Dejan Vidovic, Lucy Kathryn Helyer, Sylvia Pasternak, Carman Anthony Giacomantonio

**Affiliations:** ^1^ Division of General and Gastrointestinal Surgery, Department of Surgery, Faculty of Medicine, Dalhousie University, Halifax, NS, Canada; ^2^ Department of Pathology, Faculty of Medicine, Dalhousie University, Halifax, NS, Canada

**Keywords:** interleukin 2, IL2, BCG, intralesional, intratumoral, melanoma, in-transit melanoma

## Abstract

Cutaneous melanoma is relatively common with increasing incidence and significant mortality. While the mainstay of therapy is surgical, patients with stage III and IV disease fare poorer than those with early-stage disease and often benefit from adjuvant therapies. While systemic immunotherapy has changed the landscape of melanoma treatment, for some patients systemic toxicities related to these treatments prohibit successful administration or completion of therapy. Moreover, it is becoming increasingly evident that nodal, regional, and in-transit disease appears to be resistant to systemic immunotherapy relative to responses observed in distant metastatic disease sites. In this scenario, intralesional immunotherapies may offer benefit. In this case series, we describe the use of intralesional IL-2 and BCG at our institution in ten patients with in-transit plus or minus distant cutaneous metastatic melanoma over the last twelve years. All patients received intralesional IL2 and BCG. Both treatments were very well tolerated with only grade 1/2 adverse events. In our cohort, complete clinical response was 60% (6/10), progressive disease in 20% (2/10), and no response in 20% (2/10) of patients. The overall response rate (ORR) was 70%. The median overall survival was 35.5 months and mean overall survival 43 months in this cohort. Herein we further highlight the clinical, histopathological, and radiological course of two complete responders, showing evidence of an abscopal effect with resolution of distant untreated metastasis. Together, this limited data supports the safe and effective use of intralesional IL2 and BCG for the treatment of metastatic or in-transit melanoma in this challenging patient cohort. To our knowledge, this is the first formal study to report on this combination therapy for the treatment of melanoma.

## Introduction

Cutaneous melanoma is a relatively common cancer. The incidence is increasing yearly at a rate faster than any other form of cancer (1, 2). It also carries high mortality, as approximately 10,000 patients will die of melanoma this year in the United States alone (1). This represents an average 20.4 years of potential life lost due to melanoma in the United States (1).

The prognosis of melanoma once diagnosed is highly dependent on initial staging. Most patients present with localized disease; however, a minority present with regional (9%) or metastatic (4%) disease. For patients with stage I or II localized disease, 5 year melanoma-specific survival ranges between 99% and 82%, respectively (3). The presence of any nodal metastasis, however, is a poor prognostic factor, dropping the 5-year survival to between 32% and 84%, depending on the nodal tumor burden (3).

For early-stage disease, surgical management is sufficient. However, in patients with stage III disease (any nodal metastases or in-transit disease) or greater, prognosis tends to worsen relative to the extent of disease. Immunotherapy has significantly impacted outcomes for stage III and higher melanoma patients leading to guidelines recommending the use of immunotherapies including systemic and intralesionally administered immunotherapies for these patients (1). Current immunotherapy strategies capitalize on the inherent immunogenicity of melanoma driving established anti-tumor immunity towards a reduction and ideally clearance of all tumor burden. However, systemic immunotherapy can be associated with significant immunotherapy-related adverse events, which in some advanced melanoma trials occurs in between 30-50% of patients (4–10). To minimize this risk, when appropriate, intralesional delivery of immunotherapy is chosen over the systemic route. Currently, this strategy is generally limited to metastasis involving skin and subcutaneous tissues (with or without distant systemic metastasis).

A number of different intralesional immunotherapies have been studied in the treatment of metastatic melanoma, such as interleukin-2 (IL2), Bacillus Calmette-Guerin (BCG), checkpoint inhibitors, and oncolytic viruses (11), all to variable degrees of success. Over 40 years ago, BCG was the first intralesional immunotherapy to be used for melanoma. Pioneering American surgical oncologist Donald Morton reported lesion response rates of up to 90% in metastatic melanoma. Of note, he reported evidence of abscopal responses in approximately 17% of lesions (12). In follow-up studies where BCG was administered as an adjuvant post-surgical intervention, those who received BCG remained disease-free at 2 years at twice the incidence of the control group (13), further suggesting generation of systemic antimelanoma immunity.

However, the use of intralesional BCG waned throughout the remainder of the 20^th^ century. Subsequent randomized controlled clinical trials did not confirm or support earlier reported findings (14, 15). Indeed, in a randomized trial conducted by Morton et al, adjuvant BCG failed to provide any benefit in melanoma (16). Additionally, use of BCG caused many patients to suffer severe side effects, including abscesses, disseminated intravascular coagulation (DIC), disseminated BCG-osis, renal failure, respiratory failure, metabolic acidosis, and in some cases, death (17–19). By the early 21^st^ century, pooled analyses revealed that BCG was able to induce approximately a 20% complete response rate in stage III+ melanoma (20), significantly less than previously reported. In a large trial in 2004, BCG showed no benefit in melanoma as an adjuvant therapy, administered *via* the multiple puncture (i.e. vaccination) technique (21). Together, these unfavorable results, coupled with the toxicity profile and advent of newer chemotherapeutics caused BCG to largely fall out of clinical use.

While other intralesional agents have achieved variable success, generation of abscopal responses has seldom been observed. For example, intralesional IL2 (iIL2), the pleiotropic CD4+ and CD8+ T cell-stimulating cytokine, is reported to produce complete clinical responses (CCR) in between 32%-69% of individuals, depending on the patient population, dosing, and treatment frequency (11). Induction of anti-tumor T cell responses at the site of injection inducing immunogenic cell death (ICD) is a plausible mechanism of action; however, anti-tumor immune memory does not appear to persist, and distant untreated metastases do not reliably respond (22). The key to abscopal responses is the ability to maintain a potent immune response, with novel (i.e., neoantigen) antigen presentation to engage the adaptive arm of the immune system while driving systemic anti-tumor immunity.

When BCG was first being investigated for use in melanoma, our limited understanding of immunology hindered any meaningful mechanistic study. The replacement of lesions with pustules, ulcers, and finally noncaseating granulomas was thought to be beneficial (23), as a site where inflammatory mediators could coalesce and promote an anti-tumor immune response at the intralesional site of injection. Indeed, recent studies in the context of bladder cancer, where BCG is still used today, identify granuloma formation as an important prognostic factor in treatment success (24).

We and others have had durable responses treating patients with local, regional and in-transit cutaneous metastasis from melanoma with iIL2 monotherapy (25), as it is approved for this indication in Canada. Unfortunately, some patients experience recurrences following clinical clearance of disease while others experience incomplete or partial responses to iIL2. In cases of recurrent disease, retreatment with single agent iIL2 may be effective. However, in our prior anecdotal experience, some patients initially experiencing partial responses may go on to have a period of stable disease during iIL2 treatment but typically eventually progress locally or with distant metastasis. In some of these cases, with local provincial approval, and patient informed consent, we have implemented a strategy of introducing an adjuvant to iIL2 immunotherapy by adding intralesional BCG, a known activator of Toll-like receptors (TLR) including TLR-2 and TLR-4 and C – type lectin – like receptor Dectin 1/2 (26), with an aim to stimulating a more effective immune response. Herein, we describe our findings in treating this select group of patients at our institution with this novel approach of using combined iIL2 and BCG therapy.

## Methods

### Ethical statement on human studies

All human studies within this manuscript were conducted in accordance with the Declaration of Helsinki. Institutional ethical review and approval was obtained for this study in accordance with the local legislation and institutional requirements. This includes the institutional approval to use intralesional IL2 for melanoma in accordance with national Canadian guidelines on the treatment of advanced melanoma (27). It also includes provincial and institutional approval for the use of intralesional BCG for patients with advanced melanoma as salvage therapy. Lastly, the patients/participants provided informed consent to receive these treatments.

### Patient selection and treatment

Patients in Nova Scotia with in-transit metastases, with and without visceral metastases are routinely referred to Surgical Oncology as part of their multidisciplinary care, either primarily or from a prior medical oncology consultation. Those with unresectable and/or in-transit disease, or those who have failed prior systemic immunotherapy, are offered iIL2 treatment. For the cohort presented herein, some of the patients included actually pre-date the availability of systemic immunotherapy with check-point inhibitors at our institution. The local treatment schedule offered consisted of biweekly intralesional administration of 0.1mL to 1.0mL (500,000 – 4,000,000 IU) of IL2 to each melanotic deposit depending on lesion dimensions, to a maximum of 12 million international units (IU) per session spread amongst different lesions. After four to six sessions, biopsies are done to assess for regression. If there was an absence of clinical response, or no regression, then intralesional BCG was added in addition to the iIL2, once every two to four weeks for one to three treatments, and response reassessed. To minimize potential complications specific to the addition of BCG (e.g., BCG-osis) we limited the BCG to one or two lesions and total dose per treatment of BCG administered to 1 x 10^6^ CFU/mL per lesion, regardless of the total number of targetable metastases.

### Chart review and data analysis

Chart review was conducted by identifying patients with in-transit metastases, with and without visceral metastases treated with iIL2 and intralesional BCG at our institution. Photographic, histological, and radiological outcomes were collected, as were treatment timelines. Survival was calculated as months alive since beginning intralesional therapy, to last follow-up or death, and a Kaplan-Meier survival curve of overall survival was generated using standard methods. To document responses, RECIST 1.1 criteria were used; briefly, complete clinical response (CCR) was defined as complete regression of tumor with no clinical evidence of disease. Partial response (PR) was defined as partial regression of disease, and stable disease (SD) was defined as neither PR nor progressive disease. Progressive disease (PD) was defined as progression of targeted lesions or appearance of new non-targeted lesions. Non-response (NR) was defined as no response to treatment. Response assessments were made in real-time at the time of clinic visit/session and as stated above, dictated further IL2 treatment or offering of BCG. Response assessments were thus mostly clinical, aided at times by biopsies (i.e. after four to six sessions) and radiographic studies such as PET-CT.

## Results

### Patient and treatment characteristics and outcomes

**Figure 1:**
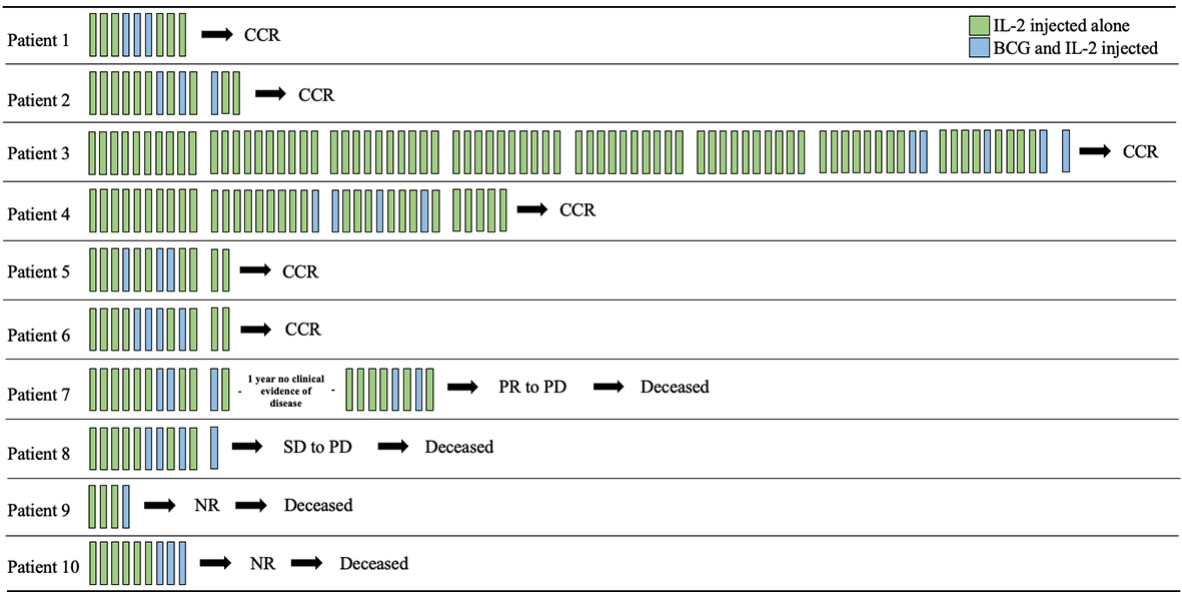
Timeline of intralesional IL2 and BCG treatments. Each bar represents a two-week period and are grouped in tens (where applicable) for ease of viewing. iIL2 treatments occur every two weeks (green bars), while BCG was added after partial response to iIL2 every 2-4 weeks for 1-4 treatments. CCR, complete clinical response; PR, partial response; PD, progressive disease; SD, stable disease; NR, no response.

We identified 10 patients treated with iIL2 and BCG at our institution since 2011, with the above inclusion criteria. The mean patient age was 75.9 (range 63-89) (**Table 1**). Seven patients were male and three were female. Six patients had metastatic disease at the time of treatment initiation, while four had locoregional disease. Mean number of iIL2 treatments was 18 (range 4-81), while mean number of BCG treatments was 3 (range 1-5). The treatment regimens for this group of patients are shown pictographically in **Figure 1**. In summary, iIL2 injections were done every two weeks, with injection of BCG every two to four weeks for one to three treatments, and clinical response was then reassessed. Mean treatment duration was 15.3 months (range 2-60). Side effects were all grade 1 and 2, and there were no grade 3 or 4 side effects for any patients (**Table 2**). iIL2 side effects generally included injection site erythema, flu-like syndrome (malaise, fatigue, chills), and fever, all self-limiting lasting between 6-48 hours. BCG side effects were similar, but for many patients also included ulceration at the injection site, a known and desired complication of BCG injection (**Table 2**).

**Table 1:** Patient characteristics, treatment characteristics and outcomes following the intralesional IL2 and BCG treatment for metastatic or locoregional melanoma.

ID Age/Race/Sex	Tumor presentation	Intralesional IL2 treatments	Intralesional BCG treatments	Treatment duration (months)	Type of responseb	Survival (months)c	Status at last follow- up
Patient 1 75/C/M	**Metastatic:** Cutaneous metastatic melanoma deposit on scalp, unknown primary	9	3	4.5	CCR	33	Alive
Patient 2 87/C/M	**Metastatic:** Melanoma on left arm, with recurrent in-transit metastases and metastases to mediastinal nodes and sternum	13	3	11	CCR	27	Alive
Patient 3 66/C/M	**Locoregional:** Melanoma on right heel, with nodal disease in ipsilateral popliteal nodes	81	5	60	CCR	108	Alive
Patient 4 80/C/F	**Locoregional:** Multiple melanoma lesions on the scalp; in-transit disease	35	5	20	CCR	45	Alive
Patient 5 81/C/F	Locoregional: Melanoma on the left arm, antecubital fossa	12	3	6	CCR	39	Alive
Patient 6 73/C/M	Metastatic: Recurrent melanoma on the right lower back with axillary lymph node metastasis	12	4	6	CCR	49	Alive
Patient 7 63/C/F	**Locoregional:** Recurrent melanoma in the left heel, with in-transit disease in left leg	201	2	34 (total)	PR to PD	38	Deceased
Patient 8 71/C/M	**Metastatic:** Melanoma in right ankle with nodal disease in right groin, inguinal nodes	11	3	5	SD to PD	16	Deceased
Patient 9 89/C/M	**Metastatic:** Melanoma in right shoulder, with dermal metastases to axilla and back	4	1	2	NR	2	Deceased
Patient 10 74/C/M	**Metastatic:** Squamous cell carcinoma in left ankle, with incidental metastatic melanoma in left groin node, unknown primary	9	3	4.5	NR	13	Deceased

a : C, Caucasian; M, male; F, female.

b : CCR, complete clinical response; PR, partial response; PD, progressive disease; SD, stable disease; NR, no response.

c : Survival measured as months alive since beginning intralesional therapy, to last follow-up or death.

^1^: With a 1-year disease-free interval between treatments 12 and 13.

**Table 2:** Side effect profile of iIL2 and intralesional BCG.

ID Age/Race/Sex	Intralesional IL2 side effects	Intralesional BCG side effects
Patient 1 75/C/M	Grade 1 injection site reaction (erythema) Grade 1 rigors/chills	Grade 1 fatigue Grade 1 fever Grade 2 injection site reaction (ulceration)
Patient 2 87/C/M	Grade 1 rigors/chills Grade 1 fatigue Grade 1 flu-like syndrome	Grade 1 rigors/chills Grade 1 fatigue Grade 2 injection site reaction (ulceration) Grade 1 flu-like syndrome
Patient 3 66/C/M	Grade 1 injection site reaction (erythema and itching) Grade 1 rigors/chills Grade 1 fever	Grade 2 injection site reaction (ulceration, erythema) Grade 2 fatigue Grade 1 flu-like syndrome
Patient 4 80/C/F	Grade 1 fatigue	Grade 2 fatigue Grade 1 fever Grade 1 chills/rigors Grade 1 flu-like syndrome Grade 1 pain (headache)
Patient 5 81/C/F	Grade 1 injection site reaction (erythema)	Grade 1 fatigue Grade 2 injection site reaction (ulceration)
Patient 6 73/C/M	Grade 1 rigors/chills	Grade 2 injection site reaction (ulceration) Grade 1 fatigue Grade 1 rigors/chills
Patient 7 63/C/F	Grade 1 fever Grade 1 rigors/chills Grade 1 flu-like syndrome (malaise)	Grade 1 rash/desquamation Grade 2 ulceration (requiring wound care)
Patient 8 71/C/M	Grade 1 fever Grade 1 rigors/chills Grade 1 nausea	Grade 1 injection site reaction (erythema, ulceration) Grade 1 fever Grade 1 rigors/chills Grade 1 nausea
Patient 9 89/C/M	Grade 1 arthritis (non-specific) Grade 1 injection site reaction (erythema)	None reported
Patient 10 74/C/M	Grade 1 fever Grade 1 rigors/chills Grade 1 fatigue	Grade 2 injection site reaction (ulceration) Grade 1 fatigue

a : C, Caucasian; M, male, F, female.

### Intralesional BCG in addition to iIL2 effectively promotes clinical response

**Figure 2:**
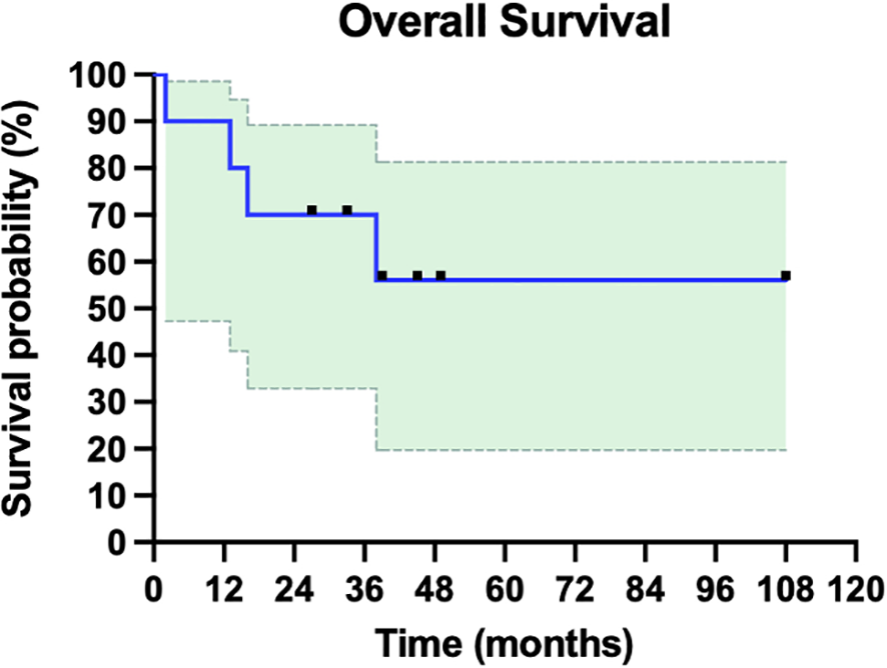
Overall survival of ten patients with metastatic or in-transit melanoma treated with iIL2 and BCG. Survival for each patient is measured as months alive since beginning intralesional therapy, to last follow-up or death. Shaded green is 95% confidence intervals. Mean overall survival was 43 months, and median overall survival in this cohort was 35.5 months.

In our cohort, the complete clinical response (CCR) following iIL2 and intralesional BCG treatment was 60% (6/10) (**Table 1**). One patient initially experienced a PR and had a one-year period of clinical response but then developed PD, leading to death (patient #7). One patient experienced stable disease (SD) with combination therapy, but eventually went on to have disease progression and death (patient #8). Thus, the overall response rate (complete responders and partial responders, by RECIST 1.1 criteria) is 70% (7/10). Notably, the number of abscopal responders (i.e. those presenting with metastatic disease and experiencing regression of non-injected lesions) was 30% (3/10). The non-response rate (NR) in this cohort, representing no response to therapy and ultimately leading to death, was 20% (2/10). The median overall survival (OS) in the total cohort was 35.5 months (range 2-108, **Figure 2**), and mean OS amongst all responders was 43 months.

### A representative sampling of complete responders

**Figure 3:**
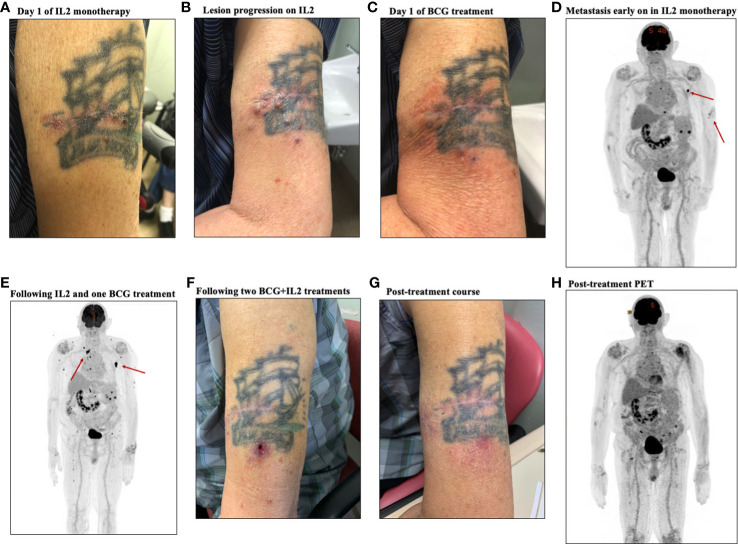
Representative clinical course of a case of a complete responder, patient #2. **(A)** Photo of primary lesion on day one of IL-2 treatment. **(B)** Photo of lesion following IL-2 monotherapy showing progression of lesions. **(C)** Photo of lesion on the day of BCG initiation, showing stable disease **(D)** PET-CT early on during IL-2 monotherapy showing extent of disease, with metastatic node highlighted. **(E)** PET-CT following treatment with IL-2 and one BCG treatment, showing metastasis. **(F)** Photo of the lesion following two BCG treatments, showing the typical ulceration seen with BCG injection. G. Photo of the treated area following treatment course, showing complete resolution. H. Post-treatment PET CT, showing reactive lymphadenopathy with no further evidence of metastatic disease and radiographic resolution of the cutaneous lesions.

**Figure 4:**
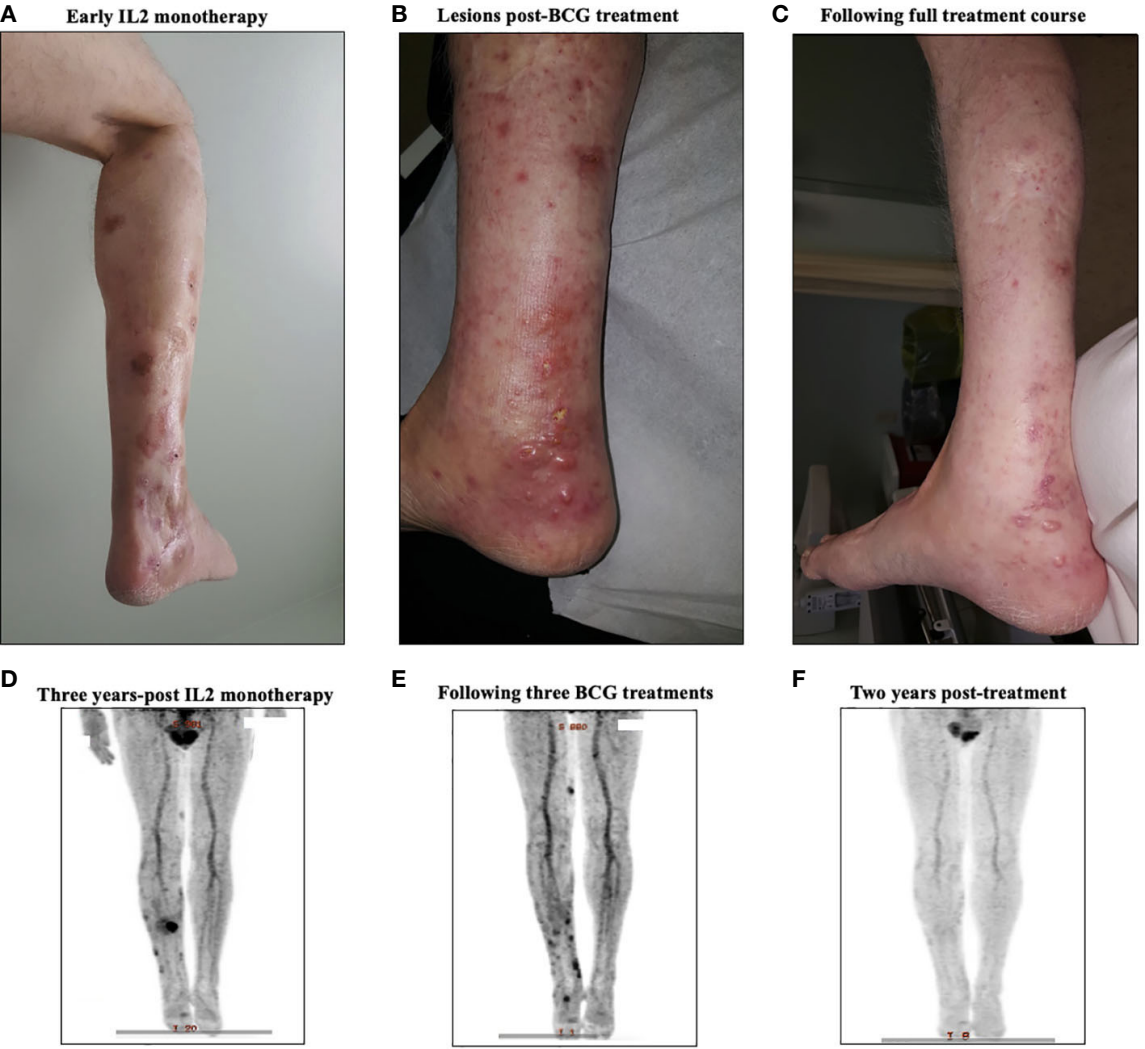
A representative case from a complete responder to intralesional IL-2 and BCG therapy. **(A)** Representative images of the treated lesion throughout IL-2 monotherapy treatment course. **(B)** PET-CT scan three years into IL-2 monotherapy showing new uptake among cutaneous lesions, signaling disease progression. **(C)** Representative image of the treated lesion following IL-2 and BCG treatment. **(D)** PET-CT scan after three BCG treatments showing regression of main lesions radiographically with new FDG uptake in distal lesions. **(E)** Representative image of the area after treatment, showing clinical resolution of BCG ulcers and in-transit metastasis. **(F)** Surveillance PET-CT two years post-treatment, showing no evidence of disease.

To highlight some of the clinical, histopathological, and radiologic characteristics of this treatment regimen, we have included clinical case data on two complete responders (**Figures 3A-H** and **Figures 4A–F**).

Patient #2, an 87-year-old male, presented to surgical oncology clinic in 2020 with a thick melanoma on the left proximal lateral arm. Wide local excision (WLE) was performed, to achieve clear margins. Soon thereafter, he developed multiple cutaneous lesions around the surgical scar (**Figure 3A**), with palpable clinical adenopathy and a positron emission tomography/computer tomography (PET-CT) scan that demonstrated metastatic disease to subcutaneous tissues and lymph nodes (**Figure 3D**). He was initially started on iIL2 monotherapy to the left arm lesions, and had six treatments approximately every two weeks, which initially was concerning for progressive disease (**Figure 3B**) but following six IL-2 treatments stabilized (**Figure 3C**). To stimulate further immunity and promote an abscopal response, BCG was added on treatment #7. A repeat PET-CT revealed radiographic stabilization of the cutaneous disease, but progressive metastasis to subcutaneous tissues and the left sternum (**Figure 3E**). Following two BCG treatments, the typical ulceration that occurs with BCG injection was observed at the cutaneous site (**Figure 3F**). Following 13 iIL2 and 3 BCG treatments, the lesions on his left arm clinically resolved (**Figure 3G**), and his post-treatment PET-CT scan revealed near complete resolution in uninjected metastatic lesions, including the bony metastasis (**Figure 3H**).

**Figure 5:**
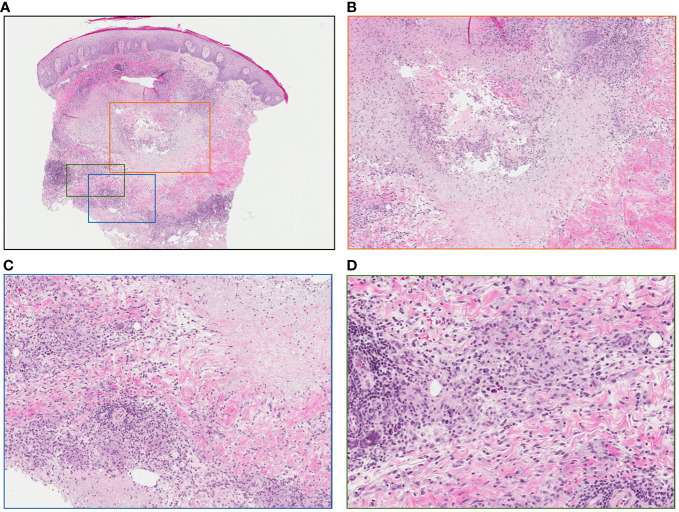
Punch biopsy of lesion following intralesional-IL-2 and BCG. **(A)** No evidence of residual melanoma, central necrosis and surrounding granulomatous reaction (low power) **(B)** Area of central necrosis (medium power) **(C)** Granulomatous reaction (medium power) **(D)** Granulomatous reaction (high power).

Patient #3, a 66-year-old male, presented in May of 2008 with a 4.3mm Breslow thickness melanoma on the right heel and underwent subsequent WLE and sentinel node biopsy of right popliteal nodes and right inguinal nodes. His pathology report ultimately came back positive for melanoma in the right popliteal nodes. He then underwent a right popliteal node dissection in Sept of 2008, which revealed 1 of 5 nodes collected as positive. Clinically and radiographically, however, he had no evidence of disease, and was closely monitored until April of 2009 when he noticed a hardened, tender lump on the posterior aspect of his right calf. The lump was excised, and pathology came back consistent with recurrent melanoma. A PET-CT scan in May 2009 revealed evidence of recurrent disease at the right malleolus, but also scattered throughout the knee joint and near the sartorius muscle on the right side representing widespread melanomatosis. He underwent isolated limb perfusion (ILP) with excision of multiple melanoma deposits and a radical groin dissection in July 2009: pathology revealed 0/9 nodes involved, but positivity in some of the resection margins on the calf lesions. He thus underwent adjuvant radiotherapy in late 2009, and had a period of stability with no clinically evidence disease until June 2010. From June 2010 until June 2012, he had seven further wide excisions of subcutaneous melanoma deposits and in-transit metastases on the right leg, all with negative margins. Given his continuously recurrent disease, in July of 2012 after multidisciplinary discussions, the decision was made to proceed with iIL2 injections to targetable recurrent lesions. He received biweekly iIL2 for four years, which caused an initial partial response and regression of lesions (**Figure 4A**). Unfortunately, he eventually experienced therapeutic escape and stable disease, followed by radiographic evidence of recurrence even on iIL2 (**Figure 4B**). In November 2016, in hopes of stimulating more robust immunity, intralesional BCG was added to the regimen, causing typical ulceration and a significant inflammatory response at the site of injection (**Figure 4C**). After three BCG treatments in total, there was clinical and radiological evidence of regression of larger sites of disease (**Figure 4D**). BCG was thus continued, and following treatment, there was no obvious clinical sign of disease (**Figure 4E**). Punch biopsies of the lesions taken after his last dose of BCG (August 2017) revealed no evidence of residual melanoma with only necrosis and granulomatous inflammation (**Figures 5A-D**). A surveillance PET-CT two years after his last dose of BCG revealed radiological complete response with no evidence of disease (**Figure 4F**), and clinically, he remains disease free five years after treatment.

## Discussion

Herein, we present a series of cases locoregional in-transit melanoma treated with iIL2 and BCG. Some patients also had concurrent distant metastatic disease.

Before treatment with BCG, the vast majority (7/10) of patients were partial responders to iIL2 monotherapy. Quite surprisingly, 60% of patients (**Table 1**) who partially responded to iIL2 went on to experience complete responses with the addition of BCG. As highlighted by patient #3, (**Figure 4**), responses were generally associated with development of ulcerations with or without localized pustules at the sites of injection (**Table 2**). Strikingly, we observed that 30% of patients responding to iIL2 plus BCG experience true abscopal responses wherein distant untreated lesions demonstrated complete responses. While abscopal responses have been reported in the context of intralesional BCG, responses of this magnitude have not yet been observed. It is notable that patients who did not respond (i.e., progressed) on iIL2, also did not respond to the addition of BCG.

The antitumor mechanism of action for BCG responses in disease remain poorly understood but is largely felt to be dependent on T-cells. In particular, the role of Th1 cell-mediated immunity including CD4+ T cells and CD8+ cytotoxic T lymphocytes (CTLs) is well known (28). More recently, some authors have attempted to identify more precise mechanisms behind the regression seen with intralesional BCG treatment in melanoma. Interestingly, intralesional BCG is able to induce atypical γδ T cell upregulation, as well as local classical immune stimulation through anti-tumor chemokine and cytokine upregulation (29). Importantly, non-injected lesions also correspondingly see an increase in γδ T cell homing and may thus experience clinical responses as well. Mechanistically, it has been hypothesized that BCG injection introduces the mycobacterial antigen hyrodxymethyl-but-2-enyl-pyrophosphate (HMBPP) into the tumor microenvironment, which promotes γδ T cell homing and recognition of said antigens (29). Incidentally, host isopentenyl pyrophosphate (IPP), produced *via* aberrant tumor pathways, is recognized as HMBPP by γδ T cells (30), which may aid in both the local anti-tumor response and the abscopal response. Indeed, in the absence of any mechanistic insight, very early electron microscopy analysis identified cross-reactivity between BCG-specific antibodies and antigens on the surface of human melanoma cells (31), further suggesting the ability of BCG to promote some level of specific anti-tumor immunity.

In our cohort, there were no grade 3/4 adverse events, suggesting the systemic effects of IL2 following intralesional injection is minimized. In prior studies wherein IL2 was used intravenously, systemic side effects such as capillary leak syndrome, hypotension, pulmonary edema, and renal dysfunction have been reported (32). The half-life of intravenously delivered IL2 is notoriously short, in the range of minutes to hours (33). In early pharmacokinetic studies, serum concentrations of IL2 following subcutaneous administration remain relatively constant for approximately 8 hours, but only reflect 2% of systemically administered concentrations (34). Early studies in rabbits and pigs suggest that subcutaneous administration preferentially concentrates IL2 in lymphatics over lymph (35, 36), supporting the rationale of local injection to maximize local immune responses. There is a paucity of high-quality data examining the pharmacokinetics of intralesional IL2 in the context of in-transit or metastasic melanoma. Further, high quality dosing studies are certainly warranted, as this may provide insight into the potential for abscopal responses.

To our knowledge, combined iIL2 and BCG to treat cutaneous metastasis from cutaneous melanoma, in the manner we have described has not been previously reported. Our results reaffirm the efficacy for IL2 as first line intralesional treatment in this patient population and present strong rational to further study the role of intralesional BCG as an adjunct to IL2 in patients with extensive local/regional recurrent melanoma, in-transit melanoma or distant cutaneous metastatic melanoma.

## Data availability statement

The original contributions presented in the study are included in the article/supplementary material. Further inquiries can be directed to the corresponding author.

## Ethics statement

The studies involving human participants were reviewed and approved by NSHA Research Ethics Board. The patients/participants provided their written informed consent to participate in this study. Written informed consent was obtained from the individual(s) for the publication of any potentially identifiable images or data included in this article.

## Author contributions

DV contributed to data collection, project conception, design of this manuscript and wrote sections of the manuscript. LH also contributed to project conception and manuscript editing. SP contributed to data collection and manuscript editing. CG contributed to project conception, data collection, design of this manuscript, project funding, and wrote sections of the manuscript. All authors contributed to the article and approved the submitted version.
